# Enzymatic Fibre Modification During Production of Dissolving Wood Pulp for Regenerated Cellulosic Materials

**DOI:** 10.3389/fpls.2021.717776

**Published:** 2021-09-28

**Authors:** Pedro E. G. Loureiro, Sonia M. S. Cadete, Radina Tokin, Dmitry V. Evtuguin, Henrik Lund, Katja S. Johansen

**Affiliations:** ^1^ Technical Industries – Forest Products Application Research, Novozymes A/S, Copenhagen, Denmark; ^2^ Department of Chemistry, University of Aveiro, Aveiro, Portugal; ^3^ Department of Geosciences and Natural Resource Management, Copenhagen University, Copenhagen, Denmark

**Keywords:** Lignocellulose, Biotechnology, enzymes, dissolving pulp, viscose

## Abstract

The production of regenerated cellulosic fibres, such as viscose, modal and lyocell, is based mainly on the use of dissolving wood pulp as raw material. Enzymatic processes are an excellent alternative to conventional chemical routes in the production of dissolving pulp, in terms of energy efficiency, reagent consumption and pulp yield. The two main characteristics of a dissolving pulp are the cellulose purity and the molecular weight, both of which can be controlled with the aid of enzymes. A purification process for paper-grade kraft pulp has been proposed, based on the use of xylanases in combination with hot and cold caustic extraction, without the conventional pre-hydrolysis step before kraft pulping. This enzyme aided purification allowed the production of a dissolving pulp that met the specifications for the manufacture of viscose, < 3% xylan, > 92% ISO brightness and 70% Fock’s reactivity. Endoglucanases (EGs) can efficiently reduce the average molecular weight of the cellulose while simultaneously increasing the pulp reactivity for viscose production. It is shown in this study that lytic polysaccharide monooxygenases act synergistically with EGs in the modification of bleached dissolving pulp.

## Introduction

Regenerated cellulosic fibre is considered a sustainable alternative to cotton and synthetic textile fibres. These are commonly produced from high-purity cellulosic wood pulp, also called dissolving pulp. Although the most common end-use of dissolving pulp is in the production of viscose fibres, alternative applications include the production of cellophane and cellulose derivatives, mainly esters and ethers. Cellulosic materials have a wide range of applications in pharmaceuticals, medicine, food and beverage, inks, ceramics, construction, etc. (Liu et al., 2016). The characteristics of a dissolving pulp for viscose applications are as: (i) high purity, (ii) appropriate molecular weight and (iii) high reactivity with carbon disulphide.

A high-purity dissolving pulp has a high cellulose content and a low content of hemicelluloses (<5% w/w), lignin and extractives. Cellulose purity is the key difference between paper-grade and dissolving-grade pulps. Harsh chemical methods are currently used in industry to obtain a sufficiently pure cellulose material for the dissolving wood pulp market (Sixta, 2006a,b).

The pulping processes in use today for the production of dissolving wood pulp are the pre-hydrolysis kraft (PHK) and the acid sulphite cooking processes. The older acid sulphite process was the predominant wood pulping method, allowing an efficient acid degradation of both lignin and hemicelluloses in the wood chips under pressure and high temperature. However, the kraft-based pulping has become the dominant process to produce dissolving wood pulp. It is less sensitive to the extractives content in the wood, allowing both hardwood and softwoods to be used. It becomes a less costly process with an efficient chemical recovery and with hardwoods gaining more importance as a more economical wood source. In addition, during the last decade, many paper-pulp producers have considered expensive conversion projects of their kraft mills to enter the dissolving pulp market, with the added possibility for a flexible switch between paper and dissolving pulp production. A key step for such conversion is the use of a pre-hydrolysis step at low pH and high temperature and pressure targeting the removal of hemicelluloses before the alkaline kraft pulping (Sixta, 2006b; Duan et al., 2015; Chen et al., 2016; Liu et al., 2016). The pre-hydrolysis intensity and the kraft delignification thus need to be optimised in a two-stage process, in order to maximise hemicelluloses and lignin removal while minimising losses in cellulose yield. Not surprisingly, such harsh cellulose purification can lead to a rather low dissolving pulp yield, *ca.* 30–35% based on wood (Kumar and Christopher, 2017).

Apart from pulp bleaching to reach high brightness, no extra cellulose purification steps are required for the removal of residual hemicelluloses in the production of conventional dissolving pulp using the PHK process. In contrast, the sulphite process typically includes hot caustic extraction (HCE) as a purification stage in the production of standard bleached dissolving pulp for viscose fibre production (Sixta, 2006b; Syed et al., 2013; Kumar and Christopher, 2017). Cold caustic extraction (CCE) is used in the production of dissolving pulp when very high purity is required, for example for cellulose acetate production (Sixta, 2006b).

The CCE purification can be considered as a more physical purification because of the high fibre swelling which leads to the solubilisation of low-molecular-weight polysaccharides, in particular targeting hemicelluloses and including the more recalcitrant portion entrapped inside the fibre wall. The typical process conditions of a CCE stage comprise a temperature range of 20–40°C and a very high concentration of NaOH (8–10% in the liquor) which in industrial scale requires an efficient recycling process of the soda and additional washing and heat-transfer capacity considering the high temperatures in the fibreline. In contrast, the HCE process uses a lower alkalinity (0.4–1.5% NaOH) and a high temperature (95–135°C) being a chemical purification based on alkaline peeling reactions of the polysaccharides, for instance end-wise peeling starting at the reducing ends. A high purification extent with HCE is limited by fibre swelling, as well as by the negative effect on cellulose yield. HCE is typically used for sulphite pulps since the carbohydrates are not stabilised during the cooking process as happens in kraft cooking with the oxidation of the reducing-end groups (Syed et al., 2013; Arnoul-Jarriault et al., 2015; Chen et al., 2016; Liu et al., 2016).

**Figure 1:**
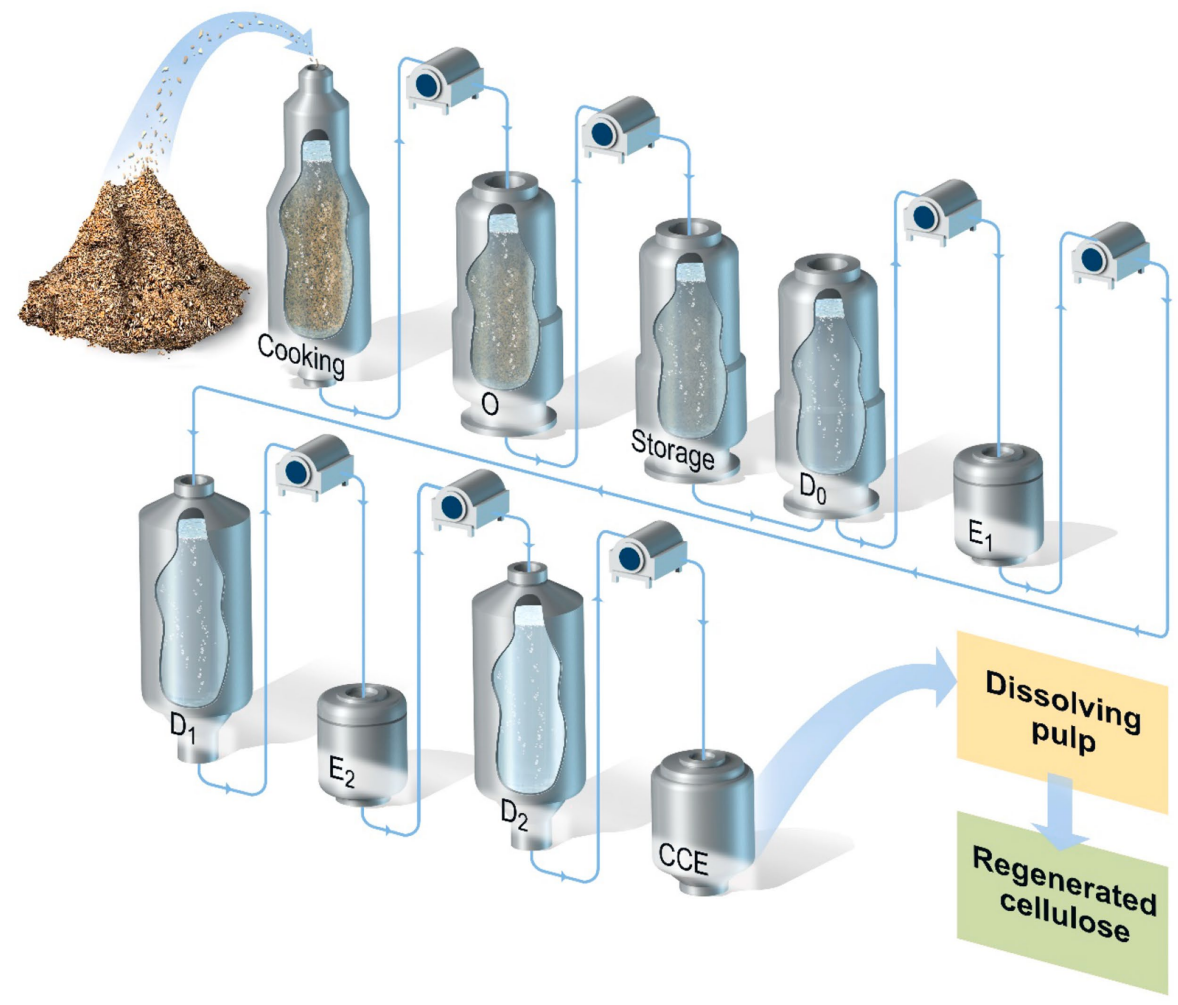
Processing of wood chips for the production of kraft dissolving pulp. The process includes the following stages after the cooking: oxygen delignification (O); storage for xylanase pre-treatment; chlorine dioxide delignification (D_0_); alkaline extraction stage 1 (E_1_); chlorine dioxide bleaching stage 1 (D_1_); alkaline extraction stage 2 (E_2_); chlorine dioxide bleaching stage 2 (D_2_); and cold caustic extraction (CCE).

Figure 1 illustrates the various stages in the production of a general kraft-based dissolving pulp, including the pulping/cooking step (pre-hydrolysis kraft) and the following multi-stage bleaching process with inter-stage washing.

Well-defined molecular weight and molecular-weight distribution of the cellulose chains are two equally important characteristics of dissolving pulp, due to their influence on pulp viscosity. The viscosity average molecular weight is commonly referred to as the intrinsic viscosity in industry. The typical intrinsic viscosity of dissolving pulps is 400–600cm^3^/g and is a key quality specification affecting downstream processing, for example in viscose fibre production plants (Sixta, 2006a).

The reactivity of a dissolving pulp defines how easily the pulp can be processed in terms of chemical usage, yield and process runnability. It is often evaluated in the lab by the Fock’s reactivity method in relation to the reaction of the pulp with carbon disulphide under predefined alkaline conditions. It is a crucial parameter for the production yield in the viscose fibre production process (Ferreira et al., 2020).

In addition to chemical processing, enzymatic processing steps with xylanases have been documented in the production of dissolving pulp. Xylanases and CCE have been used for the purification (xylan removal) and upgrading of paper-grade pulp into dissolving pulp in a number of studies (Kopcke et al., 2010; Gehmayr et al., 2011). However, it is difficult to achieve sufficient purity together with high reactivity of the dissolving pulp due to the high NaOH concentration used in CCE. This can lead to an undesirably high proportion of the different polymorphic crystalline form of cellulose II, which reduces the reactivity (Kumar and Christopher, 2017). Previous studies have shown that enzymatic treatment with endoglucanases (EGs) can improve the final reactivity of kraft dissolving pulp for viscose production (Henriksson et al., 2005; Kvarnlof et al., 2006; Ibarra et al., 2010; Miao et al., 2014).

In the present study, we have investigated the use of enzymes in a novel, greener process configuration for the production of dissolving pulp from eucalyptus wood. Eucalyptus is grown extensively as an exotic plantation species in tropical and subtropical regions and is the most widely planted hardwood in the world (Rockwood et al., 2008). A process configuration using enzymatic steps to remove hemicelluloses would eliminate the need for conventional pre-hydrolysis before kraft pulping. The hemicellulase enzymes are used to depolymerise the residual hemicelluloses in the pulp, thus creating new reducing-end groups in the polysaccharide chains (end units with aldehyde groups). The increased number of reducing groups in the hemicellulose chains triggers alkaline end-wise peeling reactions during the following HCE. It has been found that a single enzyme depolymerisation stage is insufficient for adequate hemicellulose removal, but when combined with HCE purification is synergistically enhanced (Lund et al., 2016). Considering that glucuronoxylan is the only hemicellulose in eucalypt kraft pulp, several xylanases were evaluated in the present study to improve pulp purification in subsequent conventional HCE to remove the hemicellulose. In addition, several glycoside hydrolases and esterases were screened in combination with a xylanase from GH11 family to improve cellulose purification. Hardwood xylan is a glucuronoxylan, which in the native state is partially acetylated (*O*-Acetyl-4-*O*-methyl glucuronoxylan) and also linked to lignin *via* ester linkages. Under the alkaline conditions of the kraft cooking, xylan structure is modified, for instance with deacetylation reactions and the partial demethylation of the 4-*O*-methylglucuronic acid (MeGlcA) groups attached to the xylan backbone, which are converted into hexenuronic acid (HexA) groups (Jiang et al., 2000; Sjostrom, 2006). The presence of these residual uronic groups attached to the xylan backbone are important in relation to the enzymatic cleavage of residual xylan in kraft pulps by *endo-β*-1,4-xylanases. In the case of GH11 xylanases, they require a less substituted xylan backbone compared to GH10 xylanases to be able to cleave the polysaccharide and can therefore produce longer xylosaccharides (Biely et al., 2016).

In addition to cellulose purification, enzymes also have the potential to improve the control of the average molecular weight of cellulose, allowing savings in chemical and energy demand, and increased yield (Gehmayr et al., 2011; Duan et al., 2016). Enzymes can be used either as a complement to or instead of current oxygen-based and chlorine-based bleaching technologies. To this end, we studied the effect of EGs in the control and modification of the average degree of polymerisation of cellulose, in relation to the intrinsic viscosity of the pulp. EGs were tested in combination with a relatively recently discovered class of enzymes, lytic polysaccharide monooxygenases (LPMOs), to evaluate potential synergy in enzymatic fibre modification during the production of dissolving pulp.

## Materials and Methods

### Enzymes

The enzymes studied were as: purified xylanases of family GH10 (A and B) and GH11, GH3 β-xylosidase (βX), GH5 endoglucanase, GH44 and GH45 endoglucanases, β-glucosidase, GH115 α-glucuronidase, cutinase, lipase, methyl esterase, ‘aromatic’ esterase (active in ester type lignin-carbohydrate complexes – LCC) and AA9 LPMO and a multicomponent cellulase cocktail with a GH45 endoglucanase and enriched with Novozymes Celluclast^®^.

### Pulp

Industrial oxygen-delignified (ODL) eucalyptus paper-grade kraft pulp was used. After washing in the lab, the pulp has an ISO brightness of 51.4%, kappa number 11.0 and 17.9% w/w xylan. A commercial dissolving hardwood (mixed maple and aspen) pulp produced by a pre-hydrolysis kraft process was used either as a reference or as an additional substrate for enzymatic modification.

### Pulp Treatment

The paper-grade pulp was treated using different chemical and enzymatic stages combined in different ways, in order to investigate the effect on pulp purification. GH11 and GH10 xylanases were supplemented with various auxiliary enzymes in different combinations. Control experiments were carried out under the same conditions as in the corresponding enzymatic stage but without the addition of enzymes. All the experiments were run with 10% pulp consistency in sealed polyethylene bags immersed in a temperature-controlled water bath, unless otherwise stated. After each treatment, the pulp was thoroughly washed, before the next stage or prior to pulp characterisation.

To assess the effect of enzymatic treatment on pulp intrinsic viscosity, the pulp was treated with AA9 LPMO and GH45 endoglucanase at 1.5% consistency in a Distek reactor (Distek model Symphony 7,100), under heating and continuous stirring. The pulp treatment with LPMO was supplemented with 1mM gallic acid as an electron donor. After preheating the pulp to 45°C, gallic acid was added prior to enzyme addition. The treatment was carried out at pH 5 in acetate buffer (50mM) for 25.5h, using 2mg LPMO enzyme protein (EP)/g odp (oven dried pulp) and 1.2mg EG EP/kg odp.

### Pulp Characterisation

Monosaccharide composition was determined after two-step sulphuric acid hydrolysis (modified procedure of NREL/TP-510-42,618). A set of eight calibration standards were prepared from a stock solution (0.1 g/l) composed by L-(+)arabinose D-(+)galactose D-(+)glucose, D-(+)xylose and D-(+)mannose. The calibration standards and pulp hydrolysates were analysed using high-performance anion exchange chromatography (Thermo Scientific Dionex ICS-5000+) using a CarboPac PA20 analytical column and pulsed amperometric detection. The injection volume was 2.5 μl at a flow rate of 0,380ml/min, with pressure limits of 200–5,000psi. The column temperature was 45°C, the compartment (detector) temperature 25°C and the elution was carried out using 0.2μm filtered water and a run time of 9min. The data were processed using Chromeleon software (version 7).

Pulp handsheets were prepared for ISO brightness measurements according to ISO 3688 and analysed according to ISO 2470-1. Pulp viscosity was determined according to TAPPI standard T230 om-94, and the intrinsic viscosity according to ISO 5351. Fock’s reactivity analysis was carried out with 9% NaOH to provide a measure of how much of a known amount of pulp reacts with CS_2_ as a small-scale simulation of the viscose production process (Ferreira et al., 2020). The number of aldehyde groups in the pulp (CHO content) was measured based on the reaction of the aldehydes with 2,3,4-triphenyltetrazolium chloride, as described elsewhere (Obolenskaya et al., 1991).

## Results

### Bleaching and Purification With Xylanases and HCE

The effect of an enzymatic treatment on pulp xylan content was investigated in oxygen-delignified (ODL) paper-grade eucalyptus kraft pulp. The different stages involved in the process of producing dissolving pulp are shown in Figure 1. The pulp was treated with one of two xylanases (GH11 or GH10 A), followed by a D_0_ stage and HCE, and the results are presented in Table 1. Treatment with GH11 or GH10 xylanases increased the ISO brightness of the O-X_0_-D_0_-HCE-treated pulp, but the GH11 xylanase led to a higher ISO brightness (81.9%). Treatment with GH11 xylanase resulted in the lowest level of residual xylan, 10%, which corresponds to a reduction in xylan content of ~43%.

**Table 1:** Effect of X_0_-D_0_-HCE treatment to oxygen-delignified (ODL) paper-grade kraft pulp on pulp ISO brightness and xylan content.^*^

Treatment X_0_-D_0_-HCE	ISO brightness (%)	Xylan content (% w/w)
ODL kraft paper-grade pulp	51.3	17.9
Control: X_0_-D_0_-HCE (no enzymes in X_0_)	75.8	17.2
GH11: X_0_-D_0_-HCE	81.9	10.2
GH10 A: X_0_-D_0_-HCE	79.4	11.6
Reference: Dissolving HW PHK Pulp	---	3.0–4.5

*One of two xylanases (GH11 or GH10 A) was used in the enzyme treatment. A commercial dissolving hard wood (HW) pulp was used as a reference.*

* *Conditions: X_0_ stage: 20mg EP/kg odp (GH11 or GH10 xylanase as enzyme protein); pH 4.5 (acetate buffer, 50mm), 4h, 75°C. D_0_ stage: 1.10% odp ClO_2_; pHi 2.5; 80°C; 90min and HCE stage: 6% odp NaOH; 95°C; 120min*.

As a result of the significant boost in bleaching with the xylanase in X_0_ (+6.1 ISO brightness units *versus* control), we investigated the effect of replacing the typical chlorine dioxide bleaching stage (D_1_) in Elemental Chlorine Free (ECF) bleaching with a second enzymatic stage (X_1_), together with a following HCE stage to further enhance purification. Compared to the benchmark kraft-based process in Figure 1, the xylanase is added twice: i) in the storage tower (as X_0_ stage) after the oxygen delignification (ODL, O-stage) and ii) as replacement of the chlorine dioxide stage (D1) and becoming X_1_ stage. The conventional alkaline extraction stages (E_1_ and E_2_) can be converted into HCE. When applying this extended purification sequence (X_0_-D_0_-HCE-X_1_-HCE), the xylan content in the pulp was reduced to 7.1%, which corresponds to about 60% removal, compared to the initial xylan content in the ODL kraft pulp (Table 2). Although a significant xylan removal was achieved, the purification target of <5% w/w residual xylan was not reached, as seen in the dissolving pulp used as a reference (see Table 1).

**Table 2:** Effect of X_0_-D_0_-HCE-X_1_-HCE treatment on the xylan content of oxygen-delignified (ODL) kraft paper-grade pulp.^*^

Treatment **X**_**0**_-D_0_-HCE-**X**_**1**_-HCE	Xylan content (% w/w)
Control	15.9
GH11 in X_0_ and X_1_	7.1–8.1
GH10 A in X_0_ and X_1_	8.7
X_1_: GH11, X_0_: GH11 and GH45 EG	8.2
X_1_: GH11, X_0_: GH11 and cellulases (multicomponent)	8.0

*One of two xylanases was used in the enzymatic stages X_0_ and X_1_ (GH11 or GH10 A), supplemented with GH45 EG or a multicomponent cellulase system.*

* *Conditions: X stages: 20mg EP/kg odp (as enzyme protein); pH 4.5 (acetate buffer, 50mm), 4h, 75°C. HCE stage: 6% odp NaOH; 95°C; 120min*.

### Effect of Auxiliary Enzymes on Enhanced Pulp Purification With Xylanases

During the pulp purification process, xylanase enzymes may be assisted by the addition of other biomass-degrading enzymes, which can improve the accessibility of xylan in the fibre cell wall to xylanases. The effect of cellulases, other glycoside hydrolases and esterases in combination with xylanases on pulp purification was therefore investigated. The xylanase enzymes (GH11 and GH10 A) in the X_1_ stage of the extended purification sequence (X_0_-D_0_-HCE-X_1_-E) were supplemented with auxiliary enzymes, either endoglucanase (GH45) or the multicomponent cellulolytic system consisting of a GH45 endoglucanase with Celluclast®. The enzymatic X_1_ stage was followed by an alkaline extraction stage (E stage).

The results are presented in Table 3, grouped based on the operational temperature bearing in mind the thermal stability of the enzymes. As can be seen from the table, the combined effect of the auxiliary enzymes on xylan removal was, in all cases, small or negligible compared to the single GH11 or GH10 xylanases.

**Table 3:** Combinations of enzymes applied in the X_1_ stage tested at various temperatures, with 1% pulp consistency, followed by an alkaline extraction stage (E stage) applied to the X_0_-D_0_-HCE-treated pulp.^*^

Enzyme treatment	Xylan content after X_1_-E (% w/w)
50°C	
Control	8.4
GH11 xylanase	6.8 ± 0.4
GH10 xylanase A	7.0 ± 0.4
GH3 βX	7.6 ± 0.4
GH11 xylanase + GH3 βX	6.7
GH10 xylanase A+GH3 βX	6.9 ± 0.8
GH11 xylanase+GH3 βX+GH10 xylanase A	6.2
GH11 xylanase+GH3 βX+GH5 EG	6.7 ± 0.2
GH11 xylanase+GH3 βX+GH5 EG+GH44 EG	7.0 ± 0.6
GH11 xylanase+GH3 βX+GH5 EG+multicomponent cellulases	6.4 ± 0.3
GH11 xylanase+GH3 βX+GH5 EG+GH3 β-glucosidase	6.8 ± 0.2
GH11 xylanase+GH3 βX+GH5 EG+GH45 EG	6.7 ± 0.1
GH11 xylanase+GH3 βX+‘aromatic’ esterase (LCC active)	6.6 ± 0.7
GH11 xylanase+GH3 βX+methyl esterase	6.5
GH11 xylanase+GH3 βX+cutinase	6.4 ± 0.5
GH11 xylanase+GH3 βX+lipase	6.6 ± 0.3
75°C	
Control	8.9 ± 0.1
GH11 xylanase	6.9 ± 0.6
GH10 xylanase A	8.6 ± 0.8
GH11 xylanase+GH10 xylanase A	6.7 ± 0.4
GH11 xylanase+GH5 EG+GH3 β-glucosidase	6.6 ± 0.3
GH11 xylanase+GH115 α-glucuronidase	6.8 ± 0.4
GH11 xylanase+cutinase	6.2 ± 0.1
GH11 xylanase+lipase	6.3 ± 0.3
90°C	
Control	7.9 ± 0.1
GH11 xylanase	5.2
GH10 xylanase B	5.6
GH11 xylanase + GH10 xylanase B	5.3 ± 0.2

*LCC, lignin-carbohydrate complex.*

* *Conditions: X_0_-D_0_-HCE stages as described in Table* *1* *with GH11 xylanase. X_1_ stage: 0.5 odp; 0.002mg EP/mL (as enzyme protein for each enzyme); pH 4.5 (acetate buffer, 50mm), 4h, 75°C. E stage: 0.25g odp; 6% odp NaOH; 85°C; 120min*.

When using a more aggressive consortium of cellulolytic enzymes, including cellobiohydrolases, the integrity of the fibres was compromised, as seen by visually assessment, although the residual xylan content of the solid phase was still 6.4% and thus above the target of 5%. Interestingly, the performance of the xylanase GH11 was better at 90°C than at 50°C, possibly due to its thermophilic character.

### Purification With CCE

As no significant improvement in xylan removal was observed using the auxiliary enzymes, CCE using 40 or 80g/l NaOH was also studied as a form of post-treatment, in an attempt to achieve the level of purity found in dissolving-grade pulp.

**Figure 2:**
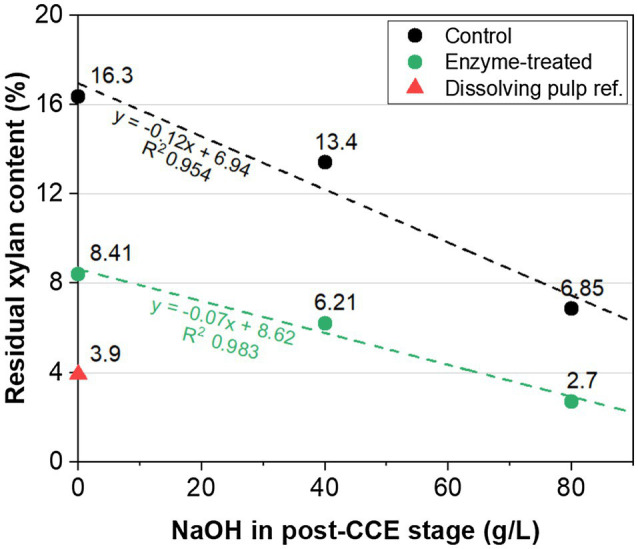
Residual xylan content in eucalyptus kraft pulp *versus* NaOH dosage in CCE. The pulp was treated with O-X_0_-D_0_-HCE-X_1_-HCE-D_1_-CCE using enzymes (green) and without any enzymes (control, black). The NaOH concentration in the post-treatment CCE was 0, 40 or 80g/l. CCE conditions: 35°C; 30min. X_0_: GH11 xylanase and cellulase; X_1_: only GH11 xylanase. The value for a commercial pre-hydrolysis kraft (PHK) hardwood dissolving pulp is shown as reference (red triangle).

Figure 2 shows the results of this investigation. It can be seen that the purity required for a viscose-grade pulp (2.7% xylan) was achieved with the enzymatic sequence with GH11 xylanase and cellulase steps, combined with CCE at 80g/l NaOH. In contrast, sufficient purity was not achieved in the control treatment without enzymes (6.8% xylan). The purity of the upgraded pulp using enzymes was also higher than the commercial hardwood dissolving pulp used as a reference (3.9% xylan). The linearity between the amount of NaOH used in CCE and the amount of xylan remaining in the pulp suggests that a significantly lower amount of NaOH is needed for the enzyme-treated pulp (~50g/l) than the extrapolated value of about 90g/l for the pulp treated without enzymes (control).

### ISO Brightness, Pulp Viscosity and Reactivity of Enzymatically Upgraded Pulp

The effect of NaOH concentration in CCE on the ISO brightness of the eucalyptus kraft pulp was also investigated. It can be seen from the results presented in Table 4 that the sequences with enzymatic steps produced very bright pulps, with ISO brightness above 92%. The increase in NaOH concentration of the CCE stage from 40 to 80g/l increased the brightness of the control-treated pulp, but not of the enzymatically treated pulp.

**Table 4:** ISO brightness of eucalyptus kraft dissolving pulp treated with the sequence O-X_0_-D_0_-HCE-X_1_-HCE-D_1_-CCE.^*^

Treatment	ISO brightness (%)
Control sequence with Post-CCE40	90.9
Enzyme sequence with Post-CCE40	92.3
Control sequence with Post-CCE80	91.8
Enzyme sequence with Post-CCE80	92.4

*The control experiment was run under the same conditions without any enzymes. Post-treatment CCE was run at 40 or 80g/l NaOH.*

* *X_0_: GH11 xylanase and cellulase (conditions as in* *Table 2* *); X_1_: only GH11 xylanase. CCE stage conditions: 35°C; 30min*.

**Figure 3:**
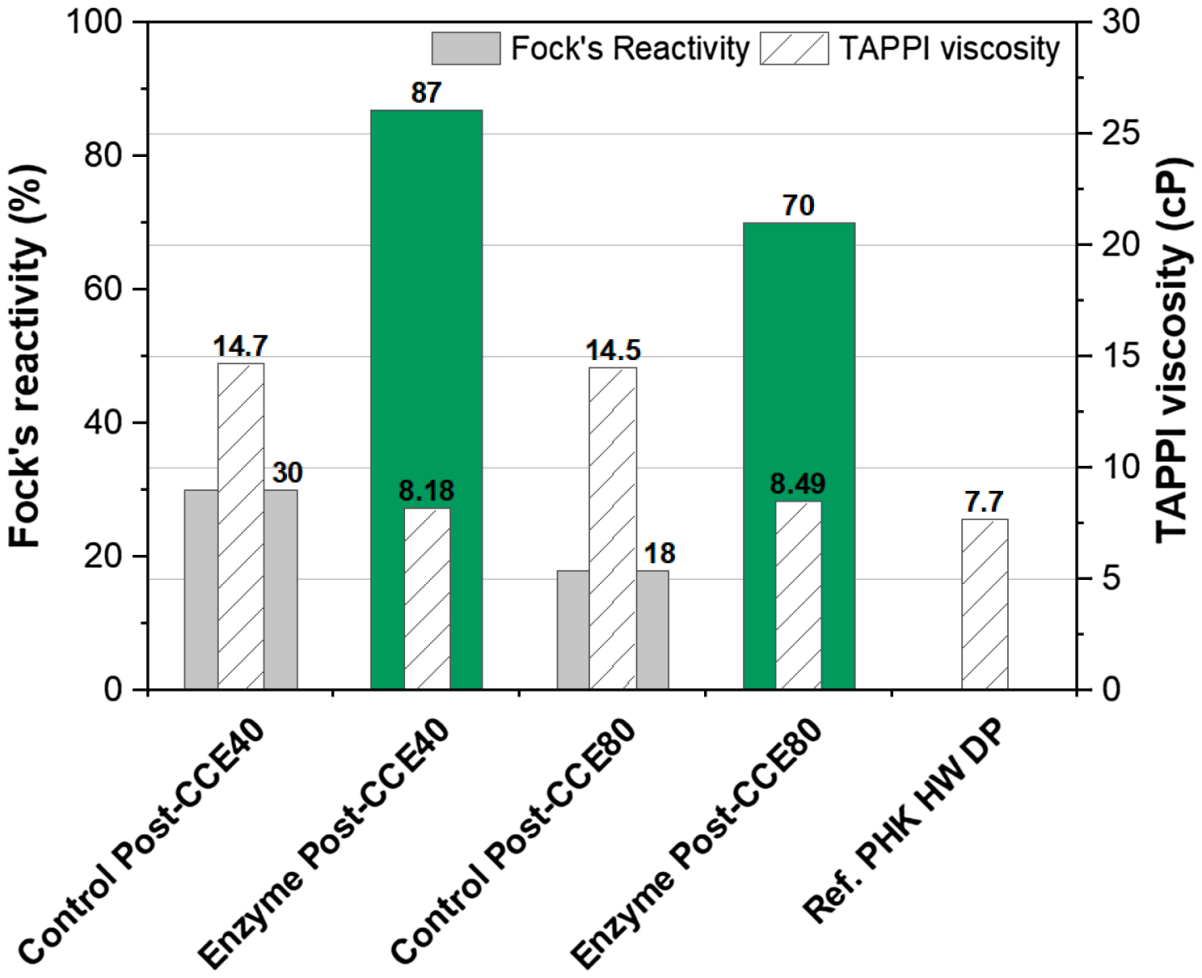
Fock’s reactivity and viscosity of eucalyptus kraft pulp after the treatment O-X_0_-D_0_-HCE-X_1_-HCE-D_1_-CCE with enzymes (green) and without enzymes (control, grey). NaOH concentration in the post-treatment CCE was 40 or 80g/l. X_0_: GH11 xylanase and cellulase; X_1_: only GH11 xylanase. A commercial PHK hardwood (HW) dissolving pulp is used as reference.

In order to assess the effect of the enzymatic treatment on pulp viscosity, the cellulase was added early in the process in the pre-bleaching stage (X_0_) rather than as post-bleaching treatment. It can be seen from Figure 3 that only one cellulase stage led to a viscosity typical of a dissolving-grade pulp (~8cP). The results also show that the pulp viscosity was efficiently reduced by the application of a cellulase to the unbleached pulp.

In terms of Fock’s reactivity, the pulp treated enzymatically with xylanase and cellulase showed much higher reactivity than the corresponding controls. Enzymatic treatment together with CCE at the higher concentration of NaOH (80g/l) led to a reactivity of 70%, compared to only18% without enzyme treatment. Increasing the NaOH dosage in CCE reduced the reactivity significantly; a greater negative effect being seen in the control than in the enzyme-treated pulp. Low reactivity resulting from high NaOH concentration in CCE is expected, as mentioned above.

### Viscosity and Reactivity of Dissolving Pulp Treated With Endoglucanase and LPMO

**Figure 4:**
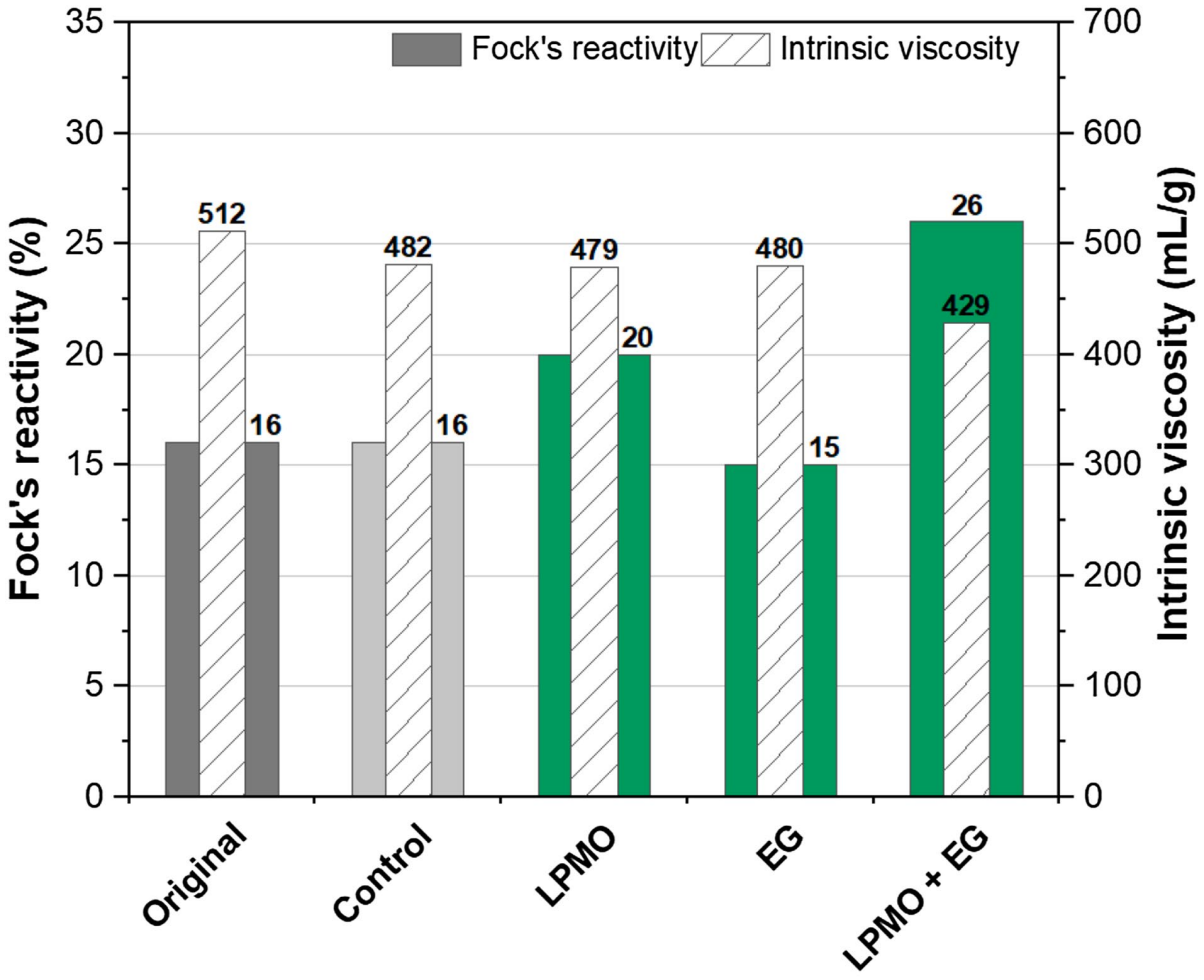
Fock’s reactivity and viscosity of a viscose-grade dissolving pulp treated with a GH45 endoglucanase (EGs) and an AA9 LPMO. The original pulp was not treated, and the control pulp was treated under the same conditions but without enzymes. The treatment was carried out at pH 5 in 50mm acetate buffer for 25.5h.

A commercial dissolving-grade pulp was treated with a GH45 endoglucanase and AA9 LPMO, either separately or in combination. The results regarding the viscosity and reactivity are illustrated in Figure 4. The commercial dissolving pulp exhibited a relatively low Fock reactivity, compared to the dissolving pulp that was produced from a paper-grade pulp using enzymes and alkaline extraction processes (Figure 3).

Both the LPMO (AA9) and the EG (GH45) had only a small effect on pulp viscosity, compared to the original pulp. A small reduction in pulp viscosity was also observed in the control experiment, which contained gallic acid. A much higher reduction in viscosity was observed after treating the pulp with the combination of LPMO and EG. A similar synergistic effect was seen in the Fock reactivity. In addition to the effects on viscosity and reactivity, the abundance of aldehyde groups in the pulps was increased slightly after treatment with LPMO and EG separately. Once again, a stronger synergistic effect was observed for the combination of these enzymes (Table 5).

**Table 5:** Aldehyde group (CHO) content in a viscose-grade dissolving pulp treated with a GH45 endoglucanase and AA9 LPMO, supplemented with 1mm gallic acid as an electron donor.

Enzyme treatment	CHO content (mmol/kg odp)
Original pulp	17.8
Control (no enzymes)	16.3
AA9 LPMO	19.8
GH45 endoglucanase	19.7
AA9 LPMO + GH45 endoglucanase	27.7

*The treatment was carried out at pH 5 in 50mm acetate buffer for 25.5 h.*

## Discussion

An alternative process for the production of kraft dissolving pulp without the use of pre-hydrolysis has been demonstrated using enzymes and alkaline extraction. It has been shown previously that GH11 family endo-xylanase hydrolyses xylan in the paper-grade pulp, but a significant amount of the hydrolysed xylan is not released from the fibre wall during enzymatic treatment (Lund et al., 2016). Therefore, subsequent alkaline treatment is required to more effectively dissolve, or further degrade and extract, the enzymatically degraded xylan. On the one hand, HCE is made more efficient as the increase in the number of reducing-end groups in xylan, generated by the prior treatment with xylanase, triggers end-wise peeling reactions in the xylan backbone as a chemical purification process. On the other hand, CCE allows pulp purification by solubilising the degraded xylan and releasing it from the swollen fibre wall (Syed et al., 2013). An important and significantly lower content of xylan was obtained in this study using a combination of enzymes and alkaline treatment, than in a previous study on the use of xylanases to remove xylan from kraft pulp (Gehmayr and Sixta, 2012).

The use of auxiliary enzymes (glycoside hydrolases and esterases) together with a xylanase did not lead to higher purity after the subsequent extraction stage. The xylan content was still too high for a dissolving-grade pulp after multicomponent cellulolytic enzymatic treatment. However, this residual hemicellulose was efficiently solubilised by CCE. Prior treatment with enzymes significantly improved the purification with CCE, which was evident from the lower NaOH concentration required. This effect was probably due to improved accessibility and the lower molecular mass of the residual hemicellulose fraction resulting from enzymatic treatment. This is an important result as high NaOH concentrations should be avoided in CCE in order to minimise the conversion of the cellulose crystal packing from native cellulose I into cellulose II. Such a conversion would negatively affect the reactivity of the pulp for the manufacturing of viscose (Syed et al., 2013).

Apart from the use of xylanases for pulp purification, their use to improve the pulp bleaching process is well known (Viikari et al., 1991; Bajpai et al., 1993). The bleach-boosting effect of xylanases was also confirmed in this study by the higher brightness values obtained. In paper-pulp bleaching, xylanase is used under controlled operational conditions to minimise reductions in yield, since xylan contributes to the papermaking properties (Schonberg et al., 2001). However, xylanase treatment can be used in the production of dissolving pulp without the risk yield losses (cellulose), as long as sufficiently pure xylanases are used.

As endoglucanases increase pulp reactivity, enzyme addition can help to reduce the negative impact of CCE on pulp reactivity if the two stages are combined in the same sequence. Previous studies have shown that a CCE stage increases the efficiency of the endoglucanase with regard to cellulose hydrolysis and, thus, also viscosity reduction (Kopcke et al., 2010; Gehmayr and Sixta, 2012). Furthermore, the use of endoglucanases for the control of pulp viscosity constitutes a greener alternative to chemical methods relying on chlorine- and oxygen-based chemicals. A single stage of enzymatic treatment with endoglucanases could reduce pulp viscosity very early in the process.

A novel method of enzymatic modification of fibres is based on the use of LPMOs. Much research has been devoted to this new class of enzymes, especially in biomass saccharification for the production of biofuels (Johansen, 2016). LPMOs may also be useful in the production of nano-cellulose (Hu et al., 2018; Koskela et al., 2019; Valenzuela et al., 2019). However, few studies have been performed on fibre modification for pulp and paper applications. This study shows that LPMOs can affect the viscosity, reactivity and aldehyde content of the dissolving pulp to a similar extent as an endoglucanase, despite their strikingly different mechanisms in cellulose depolymerisation. Furthermore, when an endoglucanase and LPMOs were applied in combination, their performance is enhanced, indicating a synergistic effect. The use of enzymes for the modification of dissolving pulps can be envisioned in both the pulp production process and in the production of regenerated cellulose.

## Conclusion

We have provided technical proof of concept regarding the application of enzymes and alkaline extraction to upgrade an ODL paper-grade eucalyptus kraft pulp into dissolving pulp, without the use of a pre-hydrolysis step before kraft pulping. Although it has not been optimised, the sequence of bleaching and purification stages, comprising O-X_0_-D_0_-HCE-X_1_-HCE-D_1_-CCE, was demonstrated to produce a dissolving pulp that met the specifications for the manufacture of viscose: 2.8% xylan; viscosity of 8.5cP; 92.4% ISO brightness; and 70% Fock’s reactivity. The promising results obtained using LPMOs combined with endoglucanases clearly show potential for enzymatic fibre modification, as demonstrated in the changes in viscosity and reactivity of a dissolving pulp.

## Data Availability Statement

The original contributions presented in the study are included in the article/supplementary material, and further inquiries can be directed to the corresponding author.

## Author Contributions

PL and HL conceived the experiments. PL and SC carried out the experiments. PL, RT and SC prepared the figures and tables. PL, SC, RT, DE and KJ co-wrote the manuscript. All authors contributed to the article and approved the submitted version.

## Funding

The publication of this work was partially funded by a grant from the Novo Nordisk Foundation (grant no. NNF17SA0027704) to KJ.

## Conflict of Interest

PL is an employee of Novozymes A/S but has not personal gain from the publication of this study.

## Publisher’s Note

All claims expressed in this article are solely those of the authors and do not necessarily represent those of their affiliated organizations, or those of the publisher, the editors and the reviewers. Any product that may be evaluated in this article, or claim that may be made by its manufacturer, is not guaranteed or endorsed by the publisher.
